# Reducing chances of COVID-19 infection by a cough cloud in a closed space

**DOI:** 10.1063/5.0029186

**Published:** 2020-10-01

**Authors:** Amit Agrawal, Rajneesh Bhardwaj

**Affiliations:** Department of Mechanical Engineering, Indian Institute of Technology Bombay, Mumbai 400076, India

## Abstract

The cough of a COVID-19 infected subject contaminates a large volume of surrounding air with coronavirus due to the entrainment of surrounding air in the jet-like flow created by the cough. In the present work, we estimate this volume of the air, which may help us to design ventilation of closed spaces and, consequently, reduce the spread of the disease. Recent experiments [P. P. Simha and P. S. M. Rao, “Universal trends in human cough airflows at large distances,” Phys. Fluids **32**, 081905 (2020)] have shown that the velocity in a cough-cloud decays exponentially with distance. We analyze the data further to estimate the volume of the cough-cloud in the presence and absence of a face mask. Assuming a self-similar nature of the cough-cloud, we find that the volume entrained in the cloud varies as $V=0.666\;c^{2}d_{c}^{3}$, where *c* is the spread rate and *d*_*c*_ is the final distance traveled by the cough-cloud. The volume of the cough-cloud without a mask is about 7 and 23 times larger than in the presence of a surgical mask and an N95 mask, respectively. We also find that the cough-cloud is present for 5 s–8 s, after which the cloud starts dissipating, irrespective of the presence or absence of a mask. Our analysis suggests that the cough-cloud finally attains the room temperature, while remaining slightly more moist than the surrounding. These findings are expected to have implications in understanding the spread of coronavirus, which is reportedly airborne.

The role of respiratory droplets in spreading the present COVID-19 pandemic, caused by SARS-CoV-2 virus particles or coronavirus, is well documented.^1–6^ There are also reports about the coronavirus being airborne.^7,8^ The role of air ejected during coughing and sneezing, and its subsequent mixing with the ambient air, is, therefore, crucial in understanding the spread of the pandemic (Fig. 1). In this context, the present work addresses the following fundamental question: when a person coughs, what is the volume of air that gets contaminated due to the cough ejected out by the person? The answer to this question is not straightforward because the surrounding air gets entrained into the cough-cloud coming out from the person’s mouth and, eventually, becomes its part; therefore, a much larger volume than initially ejected is affected by coughing. Here, we only consider the case of coughing in an environment with negligible ambient airflow. An answer to this question will help determine the maximum number of people that can be accommodated in a hospital ward and the minimum rate at which air in a room/elevator/cinema hall/car/aircraft cabin needs to be circulated to maintain freshness, reducing chances of the infection. It also helps us to calculate various thermodynamics parameters, such as temperature and relative humidity, which affect the droplet size distribution in the cloud.^9^

**FIG. 1.**
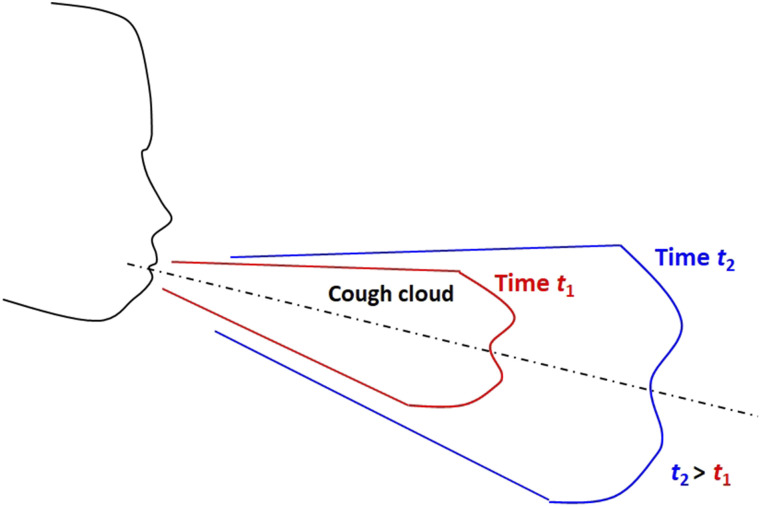
Schematic of the cough cloud generated by a human subject. The volume of the cloud increases with time due to the entrainment of the surrounding air into it.

The cloud produced by coughing can be modeled as a puff or a thermal, with initial momentum or initial buoyancy, respectively, as their driving force. Scorer^10^ was one of the first researchers to analyze thermals from a fluid dynamics perspective. He showed that the flow spreads linearly and exhibits self-similarity. Bourouiba *et al.*^11^ analyzed cough-clouds with both initial momentum and buoyancy. Given the interest in understanding the safe distance between persons and the utility of face masks during the pandemic, a number of experimental^6,12,13^ and numerical^1,2,14^ studies have recently been undertaken. For instance, results of a 3D computational model^1^ suggested that at large wind speeds varying from 4 km/h to 15 km/h, the cloud could travel up to 6 m. Vadivukkarasan *et al.*^15^ identified three instabilities, Kelvin–Helmholtz, Rayleigh–Taylor, and Plateau–Rayleigh, occurring in sequence to be the mechanism responsible for the breakup of the expelled respiratory liquid into respiratory droplets. The role of different sized droplets in spreading the disease has been examined.^7,16^ Das *et al.*^7^ recommend that the safe distance between people should be based on the distance traveled by large droplets, while the time of droplet dispersion is dictated by the dynamics of the smaller droplets. Chao *et al.*^17^ reported the number and size of respiratory droplets ejected during coughing. Busco *et al.*^18^ proposed a numerical approach for studying sneezing. In context of the use of masks, Verma *et al.*^6^ compared different types of masks and reported that well-fitted homemade masks could reduce the speed and range of the emulated cough jets significantly. The visualizations of face shields indicated that the expelled droplets can move around the visor, while an exhalation port in a mask allows a large number of droplets to pass through unfiltered, thereby reducing their effectiveness.^12^ A method to recharge an N95 mask in order to recover their filtration efficiency has been demonstrated.^19^ Dbouk and Drikakis^2^ computationally showed that the mask efficiency reduces during consecutive cough cycles and mask to face fitting is important. Li *et al.*^20^ and Wang *et al.*^21^ highlighted the spread of the pandemic during flushing of toilets. The role of weather on the spread of the disease has also been investigated.^3,22^

Thus, there is a good amount of information available on the amount of moist air and number of droplets along with their size ejected during various respiratory events (breathing, coughing, and sneezing). However, their dispersion in the surrounding air and, therefore, the possibility of transmission of the disease are still poorly understood. Keeping this gap in mind, the objective of the present work is to analyze the volume, temperature, and relative humidity of the cloud produced by coughing based on the experimental data in the literature.

First, we present a mathematical model to analyze the fluid dynamics and thermodynamics of the cough cloud. For this analysis, we use the experimental information on coughing provided by Simha and Rao.^13^ They found that jets produced by coughing, even by different subjects, can be described by the following single equation:

$$\frac{U_{c}}{U_{o}}=\exp\left(-\frac{4.763z}{d_{c}}\right),$$ (1)

where *U*_*c*_ is the front velocity, *z* is the axial coordinate, *U*_*o*_ and *d*_*c*_ are the reference velocity and length scales, respectively, used to non-dimensionalize the data, taken as the exit velocity and distance at which the velocity reduces to 1% of the exit velocity. We assume that the flow exhibits self-similarity^10,11^ and the time-averaged velocity can be described as a Gaussian function,

$$\frac{U}{U_{c}}=\exp\left(-\frac{r^{2}}{b^{2}}\right),$$ (2)

where *U* is the axial velocity, *r* is the radial coordinate, and *b* is the jet width (defined as the distance from the centerline where the axial velocity drops to *e*^−1^, where *e* is Napier’s *e* = 2.71 828, …). The jet width *b* varies linearly with *z* (i.e., *b* = *cz*, where *c* is the dimensionless spread rate of the jet—a larger spread rate implies a wider jet). A Gaussian streamwise velocity profile is already well established for free-shear flows.^23–26^

We now find the radial velocity (*V*) from the continuity equation as follows:

$$\frac{1}{r}\frac{\partial \left(rV\right)}{\partial r}+\frac{\partial U}{\partial z}=0.$$ (3)

Note the use of the cylindrical coordinate, which is clearly more appropriate than the planar coordinate system. Solving Eq. (3), we get

$$\begin{array}{ll} \left(-rV\right) & =\frac{U_{c}b^{2}}{2\;d}\left[\exp\left(-\frac{r^{2}}{b^{2}}\right)-1\right]\\ & \;+\;U_{c}cb\left[1-\exp\left(-\frac{r^{2}}{b^{2}}\right)-\left(\frac{r^{2}}{b^{2}}\right)\exp\left(-\frac{r^{2}}{b^{2}}\right)\right], \end{array}$$ (4)

where *d* ≡ *d*_*c*_/4.673.

Knowing the radial velocity allows the volume entrained into the jet to be calculated as^26^

$$\frac{d\mu }{dz}=\underset{r\to \infty }{\lim}\left(-2\pi rV\right),$$ (5)

where *μ* is the volume flow rate of the jet. The application of the above equation to a finite size room can be justified provided that the room size is sufficiently large^27^—a condition likely to be met with a person coughing in a room. Therefore,

$$\frac{d\mu }{dz}=2\pi U_{c}b\left[c-\frac{b}{2d}\right],$$ (6)

from which the volume entrained in the jet (V) can be computed as an integral of the above expression with respect to *z* and time *t*,

$$V=\int_{0}^{t}\int_{0}^{z}2\pi U_{o}\exp\left(-\frac{z}{d}\right)cz\left[c-\frac{cz}{2\;d}\right]dzdt.$$ (7)

An expression for time can be derived by integrating Eq. (1) with respect to time as follows:

$$t=\frac{d}{U_{o}}\left[\exp\left(\frac{z}{d}\right)-1\right].$$ (8)

The volume of air entrained in the cloud, therefore, varies as

$$V=\pi c^{2}z^{2}d\left[1-\exp\left(-z/d\right)\right].$$ (9)

We finally obtain a relatively simple expression for the volume entrained in the cloud,

$$\begin{array}{ll} V & =\pi c^{2}d_{c}^{2}\left(\frac{d_{c}}{4.673}\right)\left[1-\exp\left(-4.673\right)\right]\\ & =0.666\;c^{2}d_{c}^{3}, \end{array}$$ (10)

which suggests that the volume of air contained in the cloud depends only on the spread rate and distance traveled by the cloud and is independent of the initial velocity and initial volume of the cough. Note the particularly strong dependence of the volume of air contained in the cloud on the distance traveled by the cloud. The volume of the cloud equals the volume ejected plus the entrained volume, and therefore, the volume of the cloud has a dependence on the volume coughed.

We next estimate the temperature in the cough-cloud as a function of distance. For this, we solve the following equation for the conservation of energy:

$$\begin{array}{ll} & m_{a1}h_{a1}+m_{v1}h_{a1}+m_{ae}h_{ae}+m_{ve}h_{ve}-Q\\ & \;=\left(m_{a1}+m_{ae}\right)h_{a2}+\left(m_{v1}+m_{ve}\right)h_{v2}, \end{array}$$ (11)

where m is the mass and *h* is the enthalpy. Subscript “*a*” stands for air, “*v*” stands for water vapor, “*e*” stands for entrained, “1” is the upstream station (mouth), and “2” is the downstream station of interest. *Q* is the latent heat removed due to the evaporation of droplets; its effect is found to be negligible, as discussed later. The mass conservation equations are embedded in the above equation. For calculating the relative humidity, we first calculate the specific humidity *ω* = (*m*_*v*1_ + *m*_*ve*_)/(*m*_*a*1_ + *m*_*ae*_), from which the relative humidity can be found as^28^

$$RH=\frac{\omega P_{a}}{0.622P_{g}},$$ (12)

where *P*_*a*_ is the partial pressure of air and *P*_*g*_ is the partial pressure of water vapor in a saturated mixture at the given temperature.

Second, we present the results using the model described earlier. The movement of the front as a function of time is computed using Eq. (8), which utilizes the experimental data of Simha and Rao.^13^ Three cases, namely, no mask, surgical mask, and N95 mask, have been compared (Fig. 2). The front travels a large distance in the first 1 s–2 s, but then, it takes a large amount of time to cover the remaining distance. The total duration over which the cough-cloud travels is calculated to be about 5 s from Eq. (8). The use of the equation for different cases given in the work of Simha and Rao^13^ showed that for most of the cases, the cough-cloud lasts between 5 s and 8 s (Fig. 2), although the maximum time is up to 14 s, irrespective of the presence or absence of the mask (Table I). The cloud starts dissipating after this duration. This suggests that the first 5 s–8 s after coughing are particularly crucial for suspending the exhaled droplets in air. For a surgical mask, the initial velocity could be smaller while the distance traveled is comparable to the no mask case; this leads to a large time duration for the surgical mask case.

**FIG. 2.**
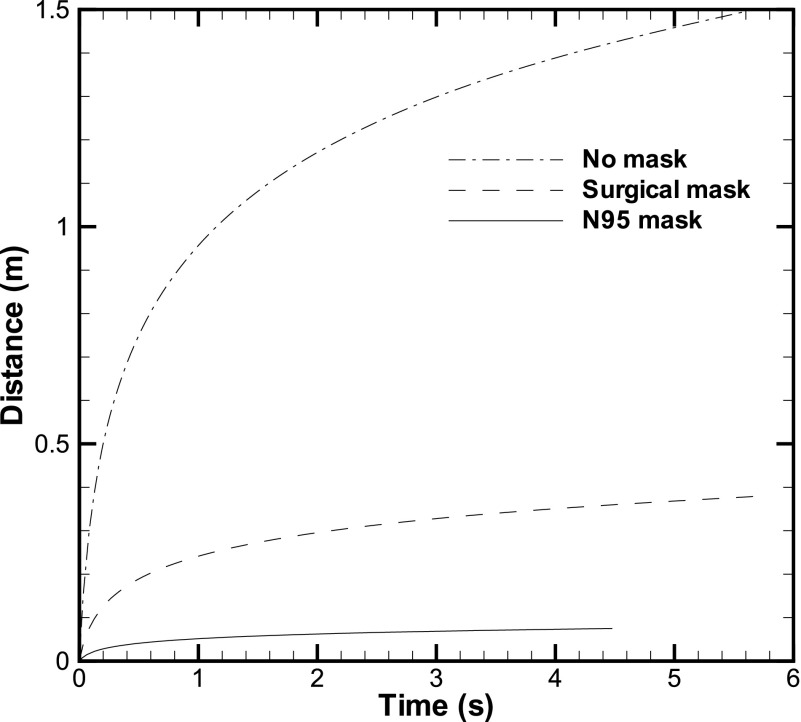
Front position as a function of time. The front position is compared for the three cases: No mask, surgical mask, and N95 mask.

**TABLE I.** Relevant parameters of the cough-cloud.

	No mask	Surgical mask	N95 mask
Distance (m) (Ref. 13)	1.5–3	0.5–1.5	0.1–0.25
Initial velocity (m/s) (Ref. 13)	3.5–6.5	1.5–2	0.4–0.8
Total time (s) [Eq. (8)]	5.7–9.8	5.7–14.2	4.5–10.0
Entrained volume (l) [Eq. (10)]	22.5–179.8	0.83–22.5	0.007–0.104

Next, we plot the lateral velocity (*V*/*U*_*c*_) as a function of the radial coordinate (*r*/*b*) inside the cloud from Eq. (4). The magnitude of the lateral velocity is clearly much smaller than the front velocity and exhibits a change in sign (Fig. 3). The lateral velocity profile is qualitatively similar to that obtained in the work of Agrawal and Prasad^26^ for other free-shear flows. We recall the reason for the change in sign:^26^ the decay of the centerline velocity leads to an outward (or positive) lateral velocity close to the centerline, while an overall increase in the jet flow rate leads to an inward (or negative) lateral velocity away from the centerline. Figure 3 clearly suggests that a large region, substantially away from the jet, is affected by the cough-cloud, and the fluid far-away is slowly entrained into the main body of the cough-cloud.

**FIG. 3.**
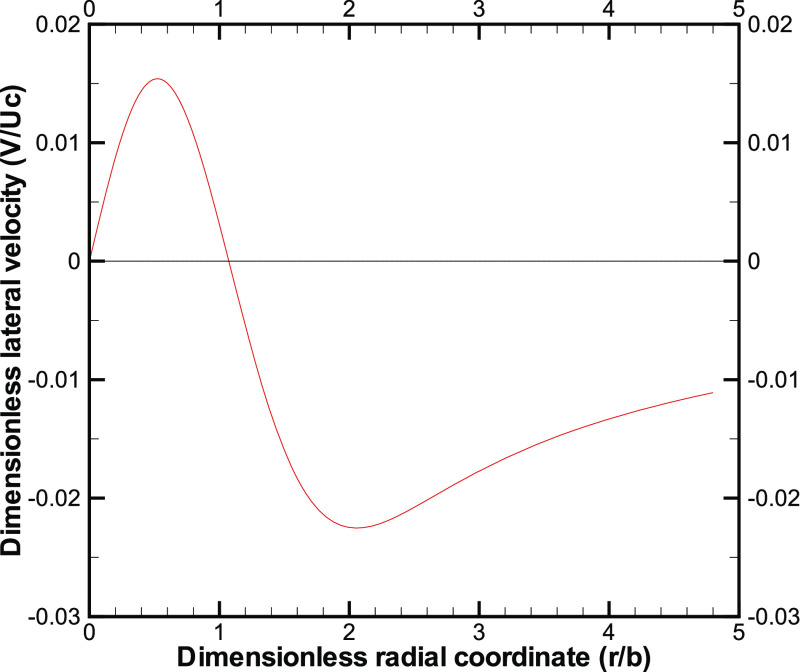
Lateral velocity as a function of the radial coordinate in the cough-cloud at 0.3 m from the origin.

The volume in the cloud increases almost quadratically with distance (Fig. 4). As the volume increases, the concentration of the droplets will drop, which will reduce the chance of infection due to a lower dose. Equation (10) suggests that for a cough with a starting velocity of 6 m/s traveling a distance of 1.5 m,^13^ we obtain the total volume of air in the cough-cloud as 0.0235 m^3^ (or 23.5 l). We have taken the spread rate of the cloud *c* = 0.1 in the above calculation, based on the data given in the work of Zhu *et al.*^29^ and other references. For comparison, the volume of air exhaled out by a person is about 1 l. The volume of air displaced by an average person of weight 68 kg is about 69 l. Therefore, the volume of air in the cough-cloud without a mask is about 23 times more than that exhaled during normal breathing and roughly equal to one-third the volume occupied by a person.

**FIG. 4.**
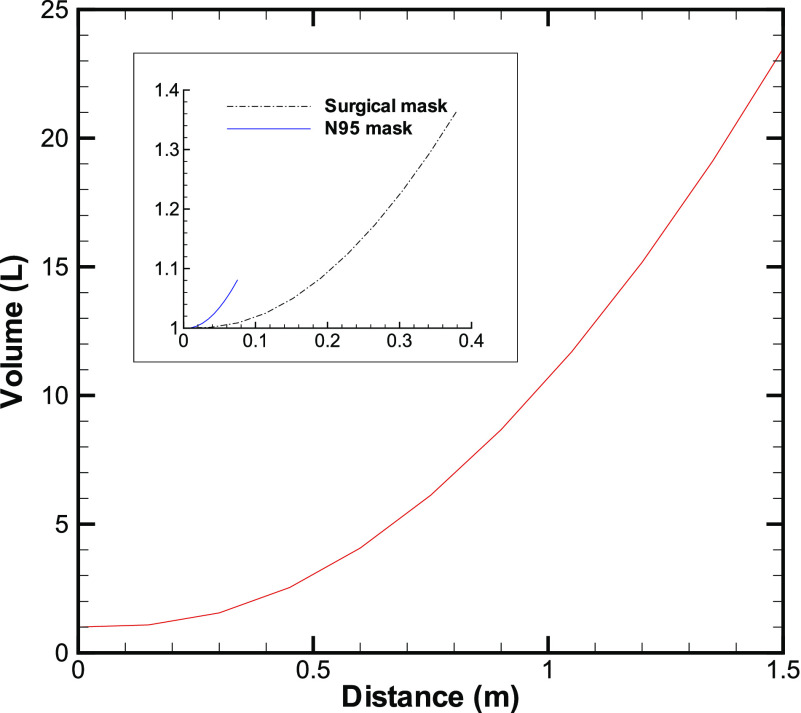
Volume of the cloud as a function of distance from the origin. The data for without a face mask are shown in the main plot, while those with the face mask are shown in the inset.

With an N95 mask on the face, the velocity reduces to about 0.52 m/s, and with the distance up to 0.23 m,^13^ we obtain the total volume of air in the cough-cloud as 0.00 108 m^3^ (or 1.08 l) (Fig. 4). A large reduction in volume is also seen with a surgical mask (Table I). A N95 mask, therefore, not only cuts the number of droplets ejected out by the person but also substantially reduces the amount of infected air produced by the person. Based on typical data,^13^ the volume of the cloud without a mask is about 7 times and 23 times larger than that with a surgical mask and an N95 mask, respectively.

The temperature of the cloud is seen to drop monotonically from the exit temperature at the origin to the room temperature (Fig. 5). For this analysis, we have assumed the volume of cough exhaled as 1 l,^11^ temperature as 33 °C,^30^ and a relative humidity of 76.7%.^30^ These representative values are taken from references reporting measurements on coughing. We assume that the cloud is ejected in a room at the temperature of 25 °C and the humidity level of either 0%, 20%, or 50%. We assume uniform adiabatic mixing of the cough-cloud with the ambient air. Note that most of the temperature drop happens between 10% and 60% of the total distance. The large amount of ambient air that mixes with the initial air ensures that the cloud ultimately attains the room temperature. The RH of the cloud exhibits a similar variation with distance from the origin (Fig. 5). The final RH is, however, slightly larger than the RH of the room (e.g., RH_*f*_ = 5.1% in dry air) suggesting that the cough-cloud is slightly more moist than the surrounding air. A slightly larger RH at *z* = 1.5 m is seen for the RH = 50% case owing to a decrease in local temperature, while the water content in the cloud is almost the same as at the exit.

**FIG. 5.**
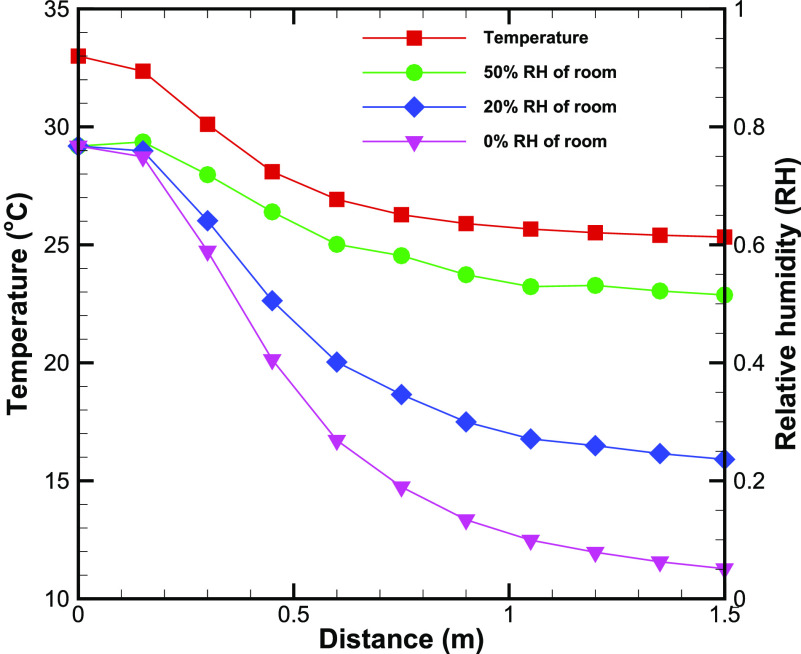
Temperature and relative humidity (RH) in the cloud as a function of distance from the origin. The plots of the RH are shown for three different values of RH of the room.

We also estimated the change in water content due to the evaporation of the droplets contained in the cloud. Toward this, we use the experimental data of Chao *et al.*^17^ reporting the variation of the droplet count as a function of droplet size produced by coughing at a distance of 10 mm from the mouth (Table II). Even if we assume that all the droplets have evaporated completely, the added water content of 4 × 10^−7^ kg is about two orders of magnitude smaller than the water content of the exhaled air. Similarly, the latent heat absorbed from the surrounding air is 0.9 J, which does not alter the temperature of moist air by more than 0.7 °C. Therefore, the estimates of temperature and RH presented above are deemed to be accurate under the set of assumptions employed.

**TABLE II.** Average droplet number count as a function of droplet size produced by coughing.^17^

Size of the droplet (*μ*m)	Count
2–4	4
4–8	55
8–16	20.4
16–24	6.7
24–32	2.5
32–40	2.4
40–50	2
50–75	2
75–100	1.4
100–125	1.7
125–150	1.6
150–200	4.4
200–250	2.5
250–500	2.1
500–1000	1.4
1000–2000	0

Finally, we compare our model predictions with the data available in the literature. Based on a dimensional analysis, researchers^10,11^ had predicted a cubic dependence of final volume on the distance traveled, which is in agreement with Eq. (10) presented here. The present analysis utilizes experimental data as the input and is expected to be more accurate; such a detailed analysis of the cough-cloud is currently unavailable. An understanding of the evolution of cloud volume helps understand various other relevant parameters, such as temperature and RH calculated in the present work.

As a further indirect validation of our result, we comment that Yin *et al.*^31^ who studied ventilation rates of 0.057 m^3^/s and 0.085 m^3^/s in a single inpatient room found that the above ventilation rates are sufficient with a person coughing in the room. With these ventilation rates, the present calculations suggest that the volume of the air equal to that of the cough-cloud can be removed in about 3 s in the worst case scenario.

In closure, the present study elucidates the mechanism of entertainment of surrounding air in the cough-cloud ejected by a human by using a mathematical model. The model utilizes available measurements of the cough-cloud. The evolving volume of the cloud is found to be independent of its initial velocity and shows a cubic dependence on the distance traveled by it. Our analysis suggests that the first 5 s–8 s after the commencement of the cough event are crucial for suspending the exhaled droplets in air and the infected air volume is around 23 times more than that ejected by coughing. The presence of a mask drastically reduces this volume and, consequently, significantly cuts down the risk of the infection to the other persons present in the room. Similarly, actions which drastically cut the distance traveled by the cloud, such as coughing into the elbow and the use of a handkerchief, can reduce the volume of a cough cloud and, therefore, the chances of dispersion of the virus. We briefly discuss the changes in temperature and relative humidity of the cloud, which could help in modeling the droplet distribution in the cloud.

While the model presented here is based on measurements of coughing, similar estimations can be made for the events of sneezing. The model can also be further extended for coughing or sneezing by several persons together or at different instances, interacting together in a same room. Similarly, the model can be extended to ambient airflow along or opposite to the direction of coughing. The key to the analysis in all the above cases would be knowing the decay rate of velocity with distance from the origin.

## DATA AVAILABILITY

The data that support the findings of this study are available from the corresponding author(s) upon reasonable request.
